# Characterization of the complete mitochondrial genome of the Northern Mud Gudgeon, *Ophiocara porocephala* (Perciformes: Eleotridae) with phylogenetic implications 

**DOI:** 10.1080/23802359.2021.1889415

**Published:** 2021-03-16

**Authors:** Muhammad Hilman Fu’adil Amin, Soo Rin Lee, Bambang Irawan, Sapto Andriyono, Hyun-Woo Kim

**Affiliations:** a Industry 4.0 Convergence Bionics Engineering, Pukyong National University, Busan, Republic of Korea; b Department of Biology, Faculty of Science and Technology, Advance Tropical Biodiversity, Genomics, and Conservation Research Group, Universitas Airlangga, Surabaya, Indonesia; c Department of Marine, Fisheries and Marine Faculty, Universitas Airlangga C Campus Jl. Mulyorejo Surabaya East Java, Surabaya, Indonesia; d Department of Marine Biology, Pukyong National University, Busan, Republic of Korea

**Keywords:** Complete mitogenome, *Ophiocara porocephala*, sleeper gobies, MiSeq

## Abstract

The first mitochondrial genome of *Ophiocara porocephala* was determined by the combination of next-generation sequencing (NGS) and Sanger sequencing methods. A complete circular mitogenome of *O. porocephala* (16,529 bp) consisted of 13 protein-coding genes, 22 transfer RNAs, two ribosomal RNAs, and two non-coding regions, including a control region (D-loop) and a light strand origin of replication (O_L_). Two start codons (ATG and GTG) and four stop codons (TAG, TAA, TA–, and T–) were used in all the PCGs. Except for ND6 and eight transfer RNAs (tRNAs), all the other genes were encoded in the heavy strand. Based on phylogenetic analysis, *O. porocephala* formed a clade with three other species in the subfamily Butinae, while the other 10 made a subfamily Eleotrinae clade.

*Ophiocara porocephala* (Valenciennes, 1837) is an amphidromous fish species in the family Eleotridae, which is widely distributed in Indo-West Pacific countries. *O. porocephala* can grow up to 30 cm in length with a high economic value in Indonesia (Thacker 2011; Borkhanuddin et al. 2014). However, the recent high exploitation grows concerns about the rapid decline its numbers threatening the sustainability of the resources (Kartamihardja 2017). Despite its wide distribution, little has been known about the genetic structure of *O. porocephala*, which is essential for the scientific conservation and management of the resource in Indonesia. We here determined the complete mitochondrial genome sequence of *O. porocephala*, as the first mitogenome information in the genus, *Ophiocara*.

*Ophiocara porocephala* was collected from brackish water of Porong River, East Java, Indonesia (7°32′35.21″S, 112°50′29.97″E) in July 2019. The collected specimen was morphologically identified by its distinct characteristics from its relatives, including the maxilla extending to below posterior of eyes and 38–40 scales in a lateral row (Kottelat et al. 1993). Nucleotide sequence in the COI region showed 100% identity to *O. porocephala* in the database (GenBank number MH085797.1) supporting again its identity. The specimen and its genomic DNA were deposited at both Department of Biology, Universitas Airlangga, Indonesia, and the Marine Biodiversity Institute of Korea (MABIK GR00004772). The mitochondrial DNA was isolated from the epaxial skeletal muscle of the specimen using a commercial kit (Abcam, Cambridge, UK), which was further fragmented by Covaris M220 Focused-Ultrasonicator (Covaris Inc., San Diego, CA). The library constructed using TruSeq^®^ RNA library kit V2 (Illumina, San Diego, CA) was sequenced by MiSeq platform (Illumina, San Diego, CA). Raw reads were assembled by Geneious software (Kearse et al. 2012) using the mitogenome of *Oxyeleotris lineolata* (NC026886) (Zang et al. 2016) as a reference. Several ambiguous regions were reconfirmed by Sanger sequencing method. Secondary structures of 22 transfer RNAs (tRNAs) were predicted by tRNAScan-SE software (Lowe and Chan 2016). A phylogenetic tree was constructed based on the combined 13 PCG regions from the currently known mitogenomes in the family Eleotridae using MEGA X Software (Kumar et al. 2018). *Odontobutis* spp. were used as outgroup member (Figure 1).

**Figure 1.**
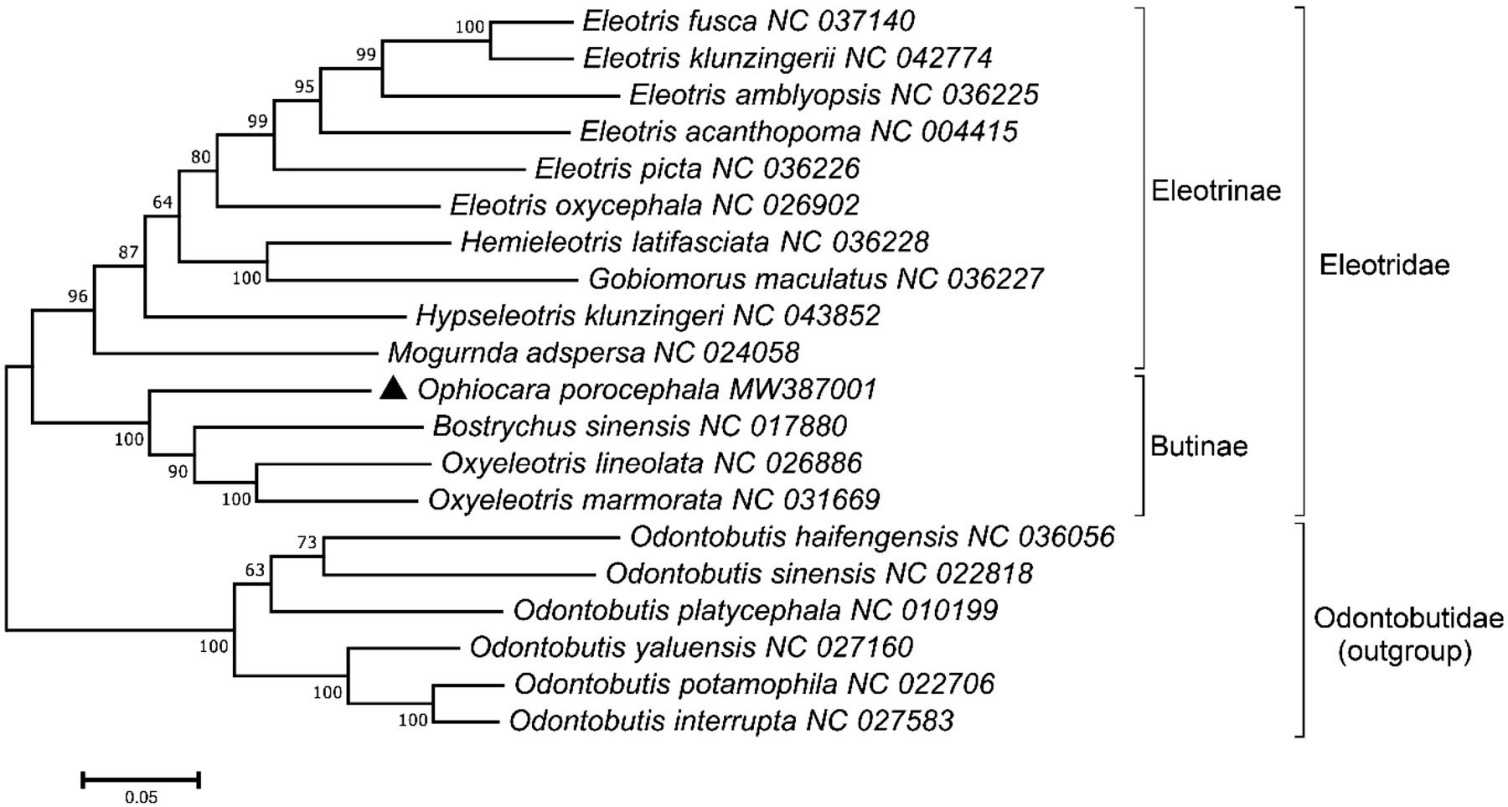
A phylogenetic tree of 14 mitogenomes in the family Eleotridae. Tree was constructed by the maximum-likelihood (ML) algorithm with 1000 bootstrap replicates. Several species in the *Odontobutis were* used as the outgroup members and the currently determined *Ophiocara porocephala* is marked by a black triangle.

The circular complete mitochondrial genome of *O. porocephala* (MW387001) was 16,529 bp in length, harboring 13 protein-coding genes (PCGs), 22 tRNAs, two ribosomal RNAs (rRNAs). Two non-coding regions, a control region (D-loop) and a light strand origin of replication (O_L_) were located between tRNA-*Pro* and tRNA-*Phe* and between tRNA*^Asn^* and tRNA*^Cys^* at the WANCY tRNA cluster, respectively (Dong et al. 2017; Andriyono et al. 2018). With the exception of the ND6 gene and eight tRNA genes, all the genes are encoded on the heavy strand. Except for COX1 gene (GTG), all the other PCGs initiate with ATG as a start codon. Either TAG or TAA was identified as a stop codon in seven PCGs, while incomplete stop codons (T– or TA–) were identified in the other six genes. The size of tRNAs ranged from 66 bp (tRNA^Cys^) to 76 bp (tRNA^Lys^). All the tRNAs were predicted to form a typical clover-leaf structure except for tRNA^Ser-GCT^, which lacked D-arm.

A phylogenetic tree constructed with 14 mitogenomes belonging to the family Eleotridae indicated that *O. porocephala* formed a Butinae subfamily clade with its sister members, including *Bostrychus sinensis* and *Oxyeleotris* spp. with 100% bootstrap value (Figure 1). Among them, *O. porocephala* was most closely related to the two species, *Oxyeleotris marmorata* (86.38%) and *Oxyeleotris lineolata* (86.31%). The other 10 species in the subfamily Eleotrinae were clustered together, distinctly from those in the Butinae, which was similar to the previous study (Alda et al. 2017). This information would provide a fundamental data for the effective management and conservation of the resource in Indonesia.

## Data Availability

The supporting data of this study are freely accessible in the GenBank with reference number MW387001 at https://www.ncbi.nlm.nih.gov/nuccore/MW387001.
